# Prevalence and Risk Factors of *Toxoplasma gondii* Infection in Domestic Cats from the Tropics of Mexico Using Serological and Molecular Tests

**DOI:** 10.1155/2012/529108

**Published:** 2012-09-11

**Authors:** Virgen J. Castillo-Morales, Karla Y. Acosta Viana, Eugenia del S. Guzmán-Marín, Matilde Jiménez-Coello, José C. Segura-Correa, A. J. Aguilar-Caballero, Antonio Ortega-Pacheco

**Affiliations:** ^1^Departamento de Salud Animal y Medicina Preventiva, CA Salud Animal, Facultad de Medicina Veterinaria y Zootecnia, Universidad Autonoma de Yucatán, Km. 15.5 Carretera, Merida-Xmatkuil, Apd. 4-116, Merida, YUC, Mexico; ^2^Laboratorio de Biología Celular, CA Biomedicina de Enfermedades Infecciosas y Parasitarias, Centro de Investigaciones Regionales, Centro de Investigaciones Regionales “Hideyo Noguchi”, Universidad Autónoma de Yucatán, Avenida Itzáes 490, 97000 Mérida, YUC, Mexico

## Abstract

The aim of this study was to determine the prevalence and risk factors associated with *Toxoplasma gondii* infection in domestic cats using an indirect-ELISA (IgM and IgG) and PCR. Samples collected from 220 cats from Merida, Yucatan, Mexico, were analyzed. Cases were reported as acute or chronic. Cases when positive to IgM and IgG and PCR were considered as reactivated chronic infection. Risk factors (sex, age, body condition, diet access to hunting, and number of cats in home) were assessed with a multivariate analysis, 75.5% (166/220) of the cats were IgM and 91.8% (202/220) IgG-seropositive and 79% were PCR-positive (173/220). Number of cats per household and low body condition score were associated with reactivated chronic infection (*P* < 0.05). It is concluded that *T. gondii* is scattered in the studied population with several periods of reinfection, and therefore an environmental contamination with infecting oocysts exists and there are intrinsic associated factors in cats that increase the risk of becoming infected.

## 1. Introduction

Toxoplasmosis is a major parasitic zoonoses of worldwide distribution caused by the intracellular protozoa *Toxoplasma gondii*. Felines, in particular domestic cats, have an important role in the epidemiology of the disease because the sexual (and asexual) reproduction takes place in them and they excrete a large number of infective and environmentally resistant oocysts [1, 2]. This parasite infects most mammals (including humans) and birds which are intermediary hosts. Among vertebrates, food animals are particularly important because they are intended for human consumption [3, 4]. Cats primarily become infected when they ingest the encysted tissue cyst from the intermediate hosts like rodents and birds. Human acquire infection when they ingest sporulated oocysts from the soil, contaminated water sources, tissue cysts from raw or undercooked meat, or transplacentally [2, 5]. Infection in cats frequently takes place as subclinical or asymptomatic disease and is very rare to develop evident clinical signs [6]. Toxoplasmosis in cats is reported worldwide with seroprevalences ranging from 60 to 90%. It is probably due to different diagnostic techniques used in each of the reported studies, cultural factors and environment of every region promoting the persistence of infective oocysts [2, 7]. In Mexico, the reported prevalence of cat toxoplasmosis varies from 9.3% to 70.8% [8–11] depending on the region. Associated risk factors for infection in cats included feeding with raw meat [8, 10], age and sex [10], and access to hunting [12]. In Mexico the seroprevalence of human toxoplasmosis is estimated to be 25% [13]. In Yucatan, Mexico, no epidemiological information about toxoplasmosis in cats is available despite the presence of the infection in humans, with a seroprevalence of 57.5% [14] and the tragic consequences associated with toxoplasmosis in AIDS patients [15].

There are several diagnostic methods for determining infection of *T. gondii*. The most widely used serological test for diagnosis of toxoplasmosis is the indirect ELISA (IgM and IgG). Moreover, the polymerase chain reaction (PCR) is a molecular test which allows detection of parasite DNA; it is highly specific and sensitive and very useful together with serological tests to differentiate the chronic, acute or reactivated infections. PCR is also an important diagnostic method when the immune system of the patient is compromised or when antibody titers have no reach threshold levels of detection [16, 17]. In domestic cats, several diseases may affect the overall health and cause immunosuppression such as infection with Bartonella spp. feline immunodeficiency virus (FIV) and feline leukemia virus (FeLV) [18, 19] that may increase the effects of acute toxoplasmosis [20, 21]. 

The information on the prevalence of toxoplasmosis in cats is useful for evaluating environmental contamination with the protozoa and to evaluate the risk it poses to public health. The aim of this study was to estimate the prevalence of toxoplasmosis in domestic cats in Merida, Yucatan, Mexico, using serological and molecular methods, and to determine possible risk factors (sex, age, diet, hunting, body condition, and number of cats per household).

## 2. Material and Methods

### 2.1. Study Area and Population

A cross-sectional study was performed in the city of Merida, Yucatan, (19° 30′ and 21° 35′ north latitude, 87° 30′ and 90° 24′ west longitude). The city's climate is characterized by warm subhumid with summer rainfall and average temperature of 26°C. The relative humidity is maximum 83% and minimum 61% with a height of 6 meters above sea level [22]. The population of interest were cats (*Felis silvestris catus*) living in Merida. The inclusion criteria were domiciled cats, of any race or sex, and older than 3 months of age.

### 2.2. Sample Size

A pilot study in 40 cats measuring IgG antibodies specific for *T. gondii* using an indirect ELISA test showed a seroprevalence of 82.5% which was used as the expected prevalence in the calculation of sample size. It was considered an infinite population of cats, a confidence level of 95% and an absolute accuracy of 5%, calculated a sample size of 222 cats [23].

### 2.3. Collection of Samples

A total of 231 blood samples (1–3 mL) were taken by puncturing the jugular vein and deposited in two vacutainer tubes, with and without anticoagulant, the first for the PCR test and the second for serological testing. DNA extraction was performed with the commercial kit DNeasy Blood and Tissue (QIAGEN, cat no. 69 506). Before extraction, a prelysis of blood was conducted as suggested by Jalal et al. [24]. The mean volume of DNA extraction from each cat was 100 *μ*L. Samples were stored at −20°C until further PCR assay. From the collected samples, 11 were not viable for diagnostic testing (negative samples by PCR for beta-globin constitutive gene) and were discarded leaving a total of 220 samples.

### 2.4. Risk Factors

Through a questionnaire survey to each owner, information was obtained to assess the following risk factors.

*Age.* Age was determined based on the review of the animal or based on their dentition [25, 26]. The animals were considered in two groups: ≤1 year and >1 until 7 years.

*Sex.* Male or female.

*Type of diet*. Two categories were considered: cats fed only with commercial food and cats fed with commercial food and/or raw meat.

*Access to the hunting.* If the cat had any access to hunting.

*Number of cats per household*. Categorized as one or more than one cat at home. 

*Body condition*. It was divided into two groups: cats with good body condition and body condition regular to bad [27].

### 2.5. Indirect ELISA Test

The presence of specific IgM and IgG antibodies against *T. gondii* was determined separately by the use of indirect ELISA tests (Human-GmbH, Wiesbaden, GER), The technique used was adapted to that described by Figueroa et al. [28], but using anti-IgM and IgG cat antibodies labeled with horseradish peroxidase (HRP) (Santa Cruz Inc., CA, USA) on 96-well plate coated with sonicated parasite proteins from tachyzoites of *T. gondii*. Serum samples were diluted to a ratio of 1 : 100 in phosphate-buffered saline (PBS; pH 7.2). The secondary goat anti-IgG and anti-IgM cat antibody HRP labeled were used at a dilution of 1 : 5,000. Sera from cats showing high anti-IgG antibodies titer by ELISA (1 : 1024) and positive results to PCR against *T. gondii* were used as positive controls, and sera pool from 10 healthy cats previously tested by triplicate with ELISA IgM, IgG and PCR, were used as negative controls. On the basis of the indirect ELISA results, subjects were diagnosed as either positive/negative for specific IgG and IgM antibodies to *T. gondii*. The optical density (OD) was measured in a spectrophotometer at 450 nm (Multiskan Multisoft Primary EIA) and was used to compute the percent positivity (PP) using the formula mean OD (sample or negative control) divided by the mean OD value positive control multiplied by 100. Percent positivity of 15% or above was considered as positive.

### 2.6. Polymerase Chain Reaction

The PCR was performed as described by [24]. Primers Tg1 (5′-AAAAATGTGGGAATGAAAGAG-3′) and Tg2 (5′-ACGAATCAACGGAACTGTAAT-3′) that amplify a fragment of 469 base pairs (bp) from the B1 gene of *T. gondii* were used. Each PCR reaction was carried out at a final concentration of 0.4 mM dNTPs, 2.5 *μ*mol of each primer, 1.5 mM MgCl^2^, and 1U of GoTaq (PROMEGA) DNA polymerase with its corresponding colorless buffer (1X), 3 *μ*L of DNA from each cat evaluated were used and the reaction was completed in a final volume of 25 *μ*L. Amplification conditions were 95°C for 10 minutes for denaturalization, followed by 35 cycles of 94°C for 1 minute, 52°C for 30 seconds and 72°C for 1 minute, and a final extension cycle of 72°C for 7 minutes. The PCR products were analyzed by electrophoresis on agarose gels and stained with 1.8% ethidium bromide. As a positive PCR control a plasmid clone pMOSBlue/Toxo 469/3, containing a sequence of 469 pb, amplification product of Tg1 and Tg2 primers of parasite genome was used.

### 2.7. Statistical Analysis

Prevalence for each serological or molecular result and its combinations was estimated. Because of the small number of cases, Chi-square test and binomial logistic regression were carried out only for IgM, IgG and PCR positive cases. Significant risk factors, under the Chi-square tests, were further investigated using binomial logistic regression. Values were considered significant when *P* < 0.05. Odds ratio (OR) and confidence interval (CI) at 95% were also estimated. Statistical analyses were carried out using the SPSS package v 17.0 (SPSS Inc., Chicago, Il, USA).

## 3. Results

### 3.1. Prevalence

**Figure 1:**
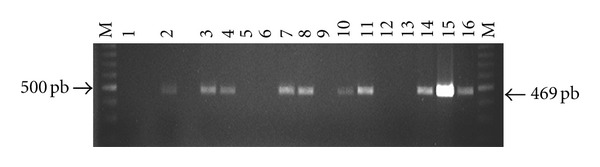
Electrophoresis in 1.8% agarose and ethidium bromide staining of amplified products with primers Tg1 and Tg2 of *T. gondii*. Lanes; (M) molecular weight marker (Promega, 100 bp DNA Ladder), (1) negative control, (2) positive control, (plasmid pMOSBlue/Toxo469/3), and samples from positive cats (3, 4, 7, 8, 10, 11, 14, 15, 16) and from negative cats (5, 6, 9, 12, 13).

Serological prevalence of *T. gondii* was 91.8% and 75.5% for IgG and IgM respectively. One hundred seventy-three samples of DNA (79%) tested amplified a 469 bp fragment corresponding to the expected size of the B1 gene sequence specific for *T. gondii.* An example of amplification is shown in Figure 1. The result of the different serological, molecular, and combination of positive and negative cases is shown in Table 1. Cases were categorized as acute when were only PCR positive or when the patient showed positive titers of IgM + PCR. Chronic cases were considered when patients were only IgG positive, IgG + PCR positive, IgM + IgG positive, or IgM + IgG + PCR positive. Positive IgM + IgG + PCR cases (61.4%), positive IgM + IgG (11.8%) and positive IgG + PCR (13.2%) were the most frequent cases. 

### 3.2. Risk Factors

The binomial logistic regression for cats with positive IgM + IgG + PCR cases showed association with body condition (fair—poor) and the presence of more than one cat at home (Table 2). However, there was no association of the variables sex and type of diet with cats infected with *T. gondii*. Also preliminary Chi-square tests showed not association of the factor cat having access to hunting and toxoplasmosis infection.

## 4. Discussion

The results of this study indicate a high rate of infection in cats with *T. gondii*, which suggests a high environmental contamination with infective oocysts. There is a wide variation in reported prevalence of feline toxoplasmosis, which is probably due to environmental, cultural, and diversity of diagnosis techniques and type of molecule measured in each study (IgA, IgM, IgG, genome, etc.). In tropical countries like Colombia, Thailand, and Brazil, reported IgG seroprevalences of toxoplasmosis in cats are 45.2% [29], 11.0% [30], and 40% [31], respectively. In some regions of Mexico the reported seroprevalence in cats is 28.8% in Colima [9], 21.9% in Mexico City [10] and 70.8% in Guadalajara [8]. This study has recorded the highest seroprevalence (91.8%) of IgG antibodies towards *T. gondii *in cats, even higher than that found in Tehran, Iran, where an overall prevalence of 63% (*n* = 100 cats) reported and from which feral cats had a 90% infection [32]. In Parana, Brazil, it was reported that 84.4% of cats were seropositive for IgG [33], which is close to the results obtained in this study. The rainfall and subtropical climate conditions with hot summers of Parana is similar to the tropical climate of the city of Merida, which is favorable to maintain viable oocysts in the environment for long periods of time.

In this study the prevalence of cats seropositive for IgM was 75.5%. In a study by Kodym et al. [34] it was reported that IgM antibodies against *T. gondii* may remain in circulation for 12–18 months, so that the titers of IgM cannot by themselves be indicators of recent infection, which is contrary to what is believed. In our study, the IgM and IgG serological status was complemented with molecular studies for a better determination of the infection status.

The percentage of acute cases of toxoplasmosis found in this study (5.9%) considering only PCR positive cases (1.8%) plus IgM, PCR positive cases (4.1%) are lower than the 8.3% found by Galván et al. [8] in Guadalajara, Mexico, considering only IgM antibodies. Acute cases are not commonly found in survey studies [35] and their presence indicates a constant dynamic of the disease and the high risk of contact of cats with the protozoan. This prevalence of cases defined as acute may depend on the chance of cats to be in contact with infecting cyts through the hunting infected prey or food provided to them by their owners, hence the importance consider adequate food for cats. Early-acute cases are those that were positive by PCR before the production of any immune response. In this study 79% of cats were positive to PCR, but only 1.81% were in the initial stage of infection where there is not production of antibodies [36] or those cats that may have compromised immune system. Suh and Joo [37] reported 5.3% of cats PCR positive to *T. gondii*. However, Lee et al. [38] more recently reported 47.2% of positive cats by using nested PCR both in South Korean conditions.

A high number of chronic cases should be expected while studying cat populations. Traditionally, cats seropositive for IgG are considered as chronically infected [6]. In this study 91.9% of cats were in the chronic stage of infection but with different serological and/or molecular status. When considering a constant exposure to the protozoa the immune response and circulation of the genome in the hot may differ giving as result the variety of diagnostic situations here reported. 

Cases when cats were IgG positives but with high levels of IgM are an indicator of revival [21]. But they also may indicate a switching from IgM to IgG weeks after infection. Reactivation in chronically infected stages involves reactivation of cysts and conversion of bradyzoites to tachyzoites and not necessarily involves a new enteroepitelial phase and excretion of oocysts [6]. However, IgM not necessarily indicates a recent infection but the outcome of the combination of different antibodies and molecular studies may be useful to confirm the chronically reactivated stage; the recirculation of the parasite in blood and serological tests indicate that there was a previous immune response.

Most cats in the chronic stage found in this study were IgM + IgG + PCR positive and are proposed to be named as chronic reactivated cases; this means that not only the parasite is circulating in the environment producing constant contact with the cats, but there are periods of reactivation with or without excretion of oocysts depending on its immune system. In chronically infected hosts with tissue cysts, these may be rupture and release bradyzoites to the circulation [39] which are eventually destroyed by the immunocompetent host or reactivate the infection in the immunosuppressed animal [40].

 It is expected that cats having access to the streets be more likely to hunt and become infected with *T. gondii*. However, in the present study no association was found between cats gaining access to the streets and hunting than those kept indoors. The nocturnal gecko *Hemidactylus frenatus* is commonly found inside all households in the area of the study which are predated by cats and ten infected; these in part may explain the results founded.

 In chronic reactivated cases, a significant protective association was found with more than one cat per household. This result is difficult to explain; however, it indicates that when living with other cats, they are less likely to be infected and reinfected with *T. gondii*, similarly as reported in cats from México city which indicates a change in environmental conditions and different immune status of cats living together [10].

 Body condition has been used as an indicator of poor nutrition in small animals and consequently a weakened immune status, increased susceptibility to disease and longer periods of parasitemia including those caused by protozoa [40, 41]. In the present study regular-to-poor body condition was significantly associated with chronic reactivated cases, indicating some degree of release and circulation of *T. gondii *oocysts. Cats with immunosuppressive diseases, poor body condition are those suffering with periods of *T. gondii *reactivation [42, 43].

 Finally, it is expected that with age, the risk of contact with the agent and infection occurs, but results from the present study indicates that cats become infected very early during their life.

 It is concluded that *T. gondii* is widely widespread in the domestic owned cat population from Merida city with various periods of reinfection and therefore there is a high environmental contamination with infective oocysts. Cats over one year old, with a poor body condition, increase the risk of becoming infected with *T. gondii*.

## Figures and Tables

**Table 1:** Type and frequency of *T. gondii* infection in cats according to serological and molecular results.

Type	IgM	IgG	PCR	*n*	%
Acute	−	−	+	4	1.8
	+	−	+	9	4.1
Chronic	−	+	−	12	5.5
	−	+	+	29	13.2
	+	+	−	26	11.8
	+	+	+	135	61.4
Negative	−	−	−	5	2.2

Total				220	100

**Table 2:** Binomial logistic regression to evaluate the association between studied variables and *T. gondii* infection cases from chronically reactivated cats. Chronically reactivated cases (positive to IgM, IgG, and PCR).

	*β*	SE	OR	CI	*P*
Age (>1–7 Years)	0.489	0.294	1.63	0.916–2.903	0.097
Cats per household (>1)	−0.714	0.292	0.49	0.276–0.869	0.015
Body condition (Regular-bad)	0.928	0.338	2.53	1.306–4.904	0.006

SE: standard error. OR: odds ratio. CI: confidence interval. *P*: *P* value.

## References

[1] Dubey JP (1995). Duration of immunity to shedding of *Toxoplasma gondii* oocysts by cats. *Journal of Parasitology*.

[2] Kravetz JD, Federman DG (2002). Cat-associated zoonoses. *Archives of Internal Medicine*.

[3] Dubey JP (2006). Comparative infectivity of oocysts and bradyzoites of *Toxoplasma gondii* for intermediate (mice) and definitive (cats) hosts. *Veterinary Parasitology*.

[4] Tenter AM, Heckeroth AR, Weiss LM (2001). Toxoplasma gondii: from animals to humans. *International Journal for Parasitology*.

[5] Dubey JP (1998). Advances in the life cycle of Toxoplasma gondii. *International Journal for Parasitology*.

[6] Lindsay DS, Blagburn BL, Dubey JP (1997). Feline toxoplasmosis and the importance of the *Toxoplasma gondii* oocyst. *Compendium on Continuing Education for the Practicing Veterinarian*.

[7] Karatepe B, Babür C, Karatepe M, Kiliç S, Dündar B (2008). Prevalence of *Toxoplasma gondii* antibodies and intestinal parasites in stray cats from Nigde, Turkey. *Italian Journal of Animal Science*.

[8] Galvan RM, Sanchez VG, Vielma MS, Soto MJ (1999). Presence of anti-Toxoplasma antibodies in humans and their cats in the urban zone of Guadalajara. *Revista da Sociedade Brasileira de Medicina Tropical*.

[9] García-Márquez LJ, Gutiérrez-Díaz MA, Correa D, Luna-Pastén H, Palma JM (2007). Prevalence of *Toxoplasma gondii* antibodies and the relation to risk factors in cats of Colima, Mexico. *Journal of Parasitology*.

[10] Besné-Mérida A, Figueroa-Castillo JA, Martínez-Maya JJ, Luna-Pastén H, Calderón-Segura E, Correa D (2008). Prevalence of antibodies against *Toxoplasma gondii* in domestic cats from Mexico City. *Veterinary Parasitology*.

[11] Dubey JP, Velmurugan GV, Alvarado-Esquivel C (2009). Isolation of *Toxoplasma gondii* from animals in Durango, Mexico. *Journal of Parasitology*.

[12] Lopes AP, Cardoso L, Rodrigues M (2008). Serological survey of *Toxoplasma gondii* infection in domestic cats from northeastern Portugal. *Veterinary Parasitology*.

[13] Gonzalo GM, Sepulveda AJ, Tapia CFR, Pérez P, Solache AG, Valdespino JL (1988). Encuesta nacional seroepidemiológica I. Diseño conceptual y metodología. *Salud Pública de México*.

[14] Velasco-Castrejón O, Salvatierra-Izaba B, Valdespino JL (1992). Seroepidemiology of toxoplasmosis in Mexico. *Salud Publica de Mexico*.

[15] Guerrero-Flores A, Vega-Ramos B (2002). Toxoplasmosis gástrica en el síndrome de inmunodeficiencia adquirida. *Review of Biomedical*.

[16] O'Neil S, Lappin M (1991). Clinical and Epidemiological aspects of FIV and Toxoplasma coinfections. *Journal of the American Animal Hospital Association*.

[17] Switaj K, Master A, Skrzypczak M, Zaborowski P (2005). Recent trends in molecular diagnostics for *Toxoplasma gondii* infections. *Clinical Microbiology and Infection*.

[18] Nutter FB, Dubey JP, Levine JF, Breitschwerdt EB, Ford RB, Stoskopf MK (2004). Seroprevalences of antibodies against *Bartonella henselae* and *Toxoplasma gondii* and fecal shedding of *Cryptosporidium* spp, *Giardia* spp, and *Toxocara cati* in feral and pet domestic cats. *Journal of the American Veterinary Medical Association*.

[19] Dubey JP, Lappin MR, Kwok OCH (2009). Seroprevalence of *Toxoplasma gondii* and concurrent bartonella spp., feline immunodeficiency virus, and feline leukemia virus infections in cats from Grenada, West Indies. *Journal of Parasitology*.

[20] Davidson MG, Rottman JB, English RV, Lappin MR, Tompkins MB (1993). Feline immunodeficiency virus predisposes cats to acute generalized toxoplasmosis. *American Journal of Pathology*.

[21] Lappin MR (1996). Feline toxoplasmosis: interpretation of diagnostic test results. *Seminars in Veterinary Medicine and Surgery-Small Animal*.

[22] Instituto Nacional de Estadística http://www.inegi.gob.mx/inegi/default.aspx.

[23] Segura CJC, Honhold N (2000). *Métodos de Muestreo Para la Producción y Salud Animal. Mérida*.

[24] Jalal S, Nord CE, Lappalainen M (2004). Rapid and sensitive diagnosis of *Toxoplasma gondii* infections by PCR. *Clinical Microbiology and Infection*.

[25] Liberg OM, Sandell D, Pontier, Turner DV (2000). Density, spatial organization and reproductive tactics in the domestic cat and other felids. *The Biology of its Behavior*.

[26] and (2004). *Geriatrics and Gerontology of the Dog and Cat*.

[27] Kroll MM, Miller PE, Rodan I (2001). Intraocular pressure measurements obtained as part of a comprehensive geriatric health examination from cats seven years of age or older. *Journal of the American Veterinary Medical Association*.

[28] Figueroa CJA, Duarte RV, Juárez AM, Luna PH, Correa D (2006). Prevalence of *Toxoplasma gondii* antibodies in rabbits (Oryctolagus cuniculus) from Mexico. *Journal of Parasitology*.

[29] Dubey JP, Su C, Cortés JA (2006). Prevalence of *Toxoplasma gondii*in cats from Colombia, South America and genetic characterization of *T. gondii* isolates. *Veterinary Parasitology*.

[30] Jittapalapong S, Nimsupan B, Pinyopanuwat N, Chimnoi W, Kabeya H, Maruyama S (2007). Seroprevalence of *Toxoplasma gondii* antibodies in stray cats and dogs in the Bangkok metropolitan area, Thailand. *Veterinary Parasitology*.

[31] Meireles LR, Galisteo AJ, Pompeu E, Andrade HF (2004). *Toxoplasma gondii* spreading in an urban area evaluated by seroprevalence in free-living cats and dogs. *Tropical Medicine and International Health*.

[32] Haddadzadeh HR, Khazraiinia P, Aslani M (2006). Seroprevalence of *Toxoplasma gondii* infection in stray and household cats in Tehran. *Veterinary Parasitology*.

[33] Dubey JP, Navarro IT, Sreekumar C (2004). *Toxoplasma gondii* infections in cats from Paraná, Brazil: seroprevalence, tissue distribution, and biologic and genetic characterization of isolates. *Journal of Parasitology*.

[34] Kodym P, Machala L, Roháčová H, Širocká B, Malý M (2007). Evaluation of a commercial IgE ELISA in comparison with IgA and IgM ELISAs, IgG avidity assay and complement fixation for the diagnosis of acute toxoplasmosis. *Clinical Microbiology and Infection*.

[35] Lappin MR, Powell CC (1991). Comparison of latex agglutination, indirect hemagglutination, and ELISA techniques for the detection of *Toxoplasma gondii*-specific antibodies in the serum of cats. *Journal of Veterinary Internal Medicine*.

[36] Dubey JP, Lappin MR, Thulliez P (1995). Long-term antibody responses of cats fed *Toxoplasma gondii* tissue cysts. *Journal of Parasitology*.

[37] Suh MD, Joo BH (1999). Polymerase chain reaction for the detection of *Toxoplasma gondii* in the blood of cats. *Korean Journal of Veterinary Research*.

[38] Lee JY, Lee SE, Lee EG, Song KH (2008). Nested PCR-based detection of *Toxoplasma gondii* in German shepherd dogs and stray cats in South Korea. *Research in Veterinary Science*.

[39] Dubey JP, Lindsay DS, Speer CA (1998). Structures of *Toxoplasma gondii* tachyzoites, bradyzoites, and sporozoites and biology and development of tissue cysts. *Clinical Microbiology Reviews*.

[40] Frenkel JK (1956). Pathogenesis of toxoplasmosis and of infections with organisms resembling Toxoplasma. *Annals of the New York Academy of Sciences*.

[41] Petersen RM, Gürtler RE, Cecere MC (2001). Association between nutritional indicators and infectivity of dogs seroreactive for Trypanosoma cruzi in a rural area of northwestern Argentina. *Parasitology Research*.

[42] Lappin MR, Gasper PW, Rose BJ, Powell CC (1992). Effect of primary phase feline immunodeficiency virus infection on cats with chronic toxoplasmosis. *Veterinary Immunology and Immunopathology*.

[43] Lappin MR, Dawe DL, Lindl PA, Greene CE, Prestwood AK (1991). The effect of glucocorticoid administration on oocyst shedding, serology, and cell-mediated immune responses of cats with recent or chronic toxoplasmosis. *Journal of the American Animal Hospital Association*.

